# Health screening of free-ranging European brown hares (*Lepus europaeus*) on the German North-Sea island Pellworm

**DOI:** 10.1186/s13028-015-0132-0

**Published:** 2015-08-04

**Authors:** Annika Posautz, Igor Loncaric, Marie Lundin, Daniel Hoffmann, Antonio Lavazza, Zsofia Kelemen, Christoph Beiglböck, Christian Walzer, Anna Kübber-Heiss

**Affiliations:** Research Institute of Wildlife Ecology, University of Veterinary Medicine Vienna, Savoyenstrasse 1, 1160 Vienna, Austria; Institute of Bacteriology, Mycology and Hygiene, University of Veterinary Medicine Vienna, Veterinärplatz 1, 1210 Vienna, Austria; Istituto Zooprofilattico Sperimentale della Lombardia e dell’Emilia Romagna, Via Bianchi 7/9, 25124 Brescia, Italy; Game Conservancy Deutschland e.V, Schloßstrasse 1, 86723 Oettingen, Germany

**Keywords:** Animals, European brown hare, Intestine, *Lepus europaeus*, Microflora, Pathology, Histopathology

## Abstract

**Background:**

A sudden decline of the European brown hare (*Lepus europaeus*) population in one of the best hunting districts for small game species in northern Germany, the German North-Sea island Pellworm, in the years 2007/08 following marked habitat changes led to the implementation of a thorough health assessment program of the population. 110 animals were collected during the normal hunting season in the years 2010 and 2011. A post-mortem examination and histopathological investigation was performed on all animals. Additionally, routine bacteriology of the small intestine and parasitology were carried out. Sera of hares were tested for European Brown Hare Syndrome (EBHS) by enzyme linked immunosorbent assay, and for *Treponema* sp. by indirect immunofluorescent test. Additional testing was performed when deemed necessary.

**Results:**

The most striking result was a shift in the intestinal bacterial flora towards Gram-negative *Enterobacteriaceae* with a predominance of either *Escherichia coli*, or *Aeromonas* sp., or a high-grade double-infection with these two pathogens with subsequent catarrhal enteritis. Additionally, a marked coccidiosis, and varying infestations with the nematode *Trichostrongylus retortaeformis* were found. The sero-prevalence for EBHS was 78.1%, and for *Treponema* 43.9%.

**Conclusions:**

The shift and decrease in diversity of the intestinal flora was the main and most consistent result found. In the authors’ opinion the change of the habitat combined with other stressors increased the animals’ sensitivity to ubiquitous bacterial species and parasites which usually would not have such fatal effects.

## Background

The European brown hare (*Lepus europaeus*) is one of the most important game animal species in Central Europe. Although hares are highly adaptable to a great number of different habitat types, a decline of this species throughout its range has been noted since the 1960s [e.g. [1–4]. It is classified as “least concern” by the International Union for Conservation of Nature (IUCN), however some countries have placed it as “near threatened” or “threatened” on their own national red list [5]; especially for some regional populations there is a growing concern. In Germany the European brown hare is listed as “endangered” [5]. Much research has been performed in regard to habitat preferences and abundance [e.g. 1, 6]. Nevertheless, it is not clear why the numbers are declining. Probably the most important threat for this species is the intensification of agriculture [4]. As Milanov [7] showed, crop harvesting operation is a source of mortality if leverets are using the crops for cover. If available the European brown hare prefers weeds and wild grasses, but in areas of agricultural intensification these foods are reduced and crop species are increasingly used as a food source [8]. Another serious threat for this species is predation, especially by foxes (*Vulpes vulpes*), which can increase the mortality rate by 50% during winter and 20% during summer time [9]. Additionally, diseases have been identified to have a high impact on the mortality [10]. In short, these include amongst others the European Brown Hare Syndrome (EBHS), a highly contagious viral disease with a reported mortality across Europe varying between 4 and 56% [11]. Pasteurellosis, also known as haemorrhagic septicemia, is caused by *Pasteurella multocida*. Although it is a very common bacterium in compromised animals the disease can lead to death within 12–48 h [12]. One of the most important causes of death in hares, with an increase in colder months, is pseudotuberculosis caused by pathogenic strains of the genus *Yersinia* [13, 14].

The hare population on the German North-Sea island Pellworm was stable for many years, with a hunting quota as high as 759 individuals in 2000. But in the years 2006/07 following massive changes to the landscape (*inter alia*: increase corn production for bioenergy) the hunting quota sank to an all-time low of 151 hares (pers. comm. Dr. Hoffmann). Considering the various reasons for a population decline, and the overall situation of the hare, it was decided to implement a health assessment of this population trying to grasp the cause in this specific case.

We report the findings of a thorough health assessment including necropsy, histopathology, parasitology, bacteriology, serology and further analysis (PCR, special stains for histology) when deemed necessary of a free-ranging island population of European brown hares. The aim of this study was to obtain an overview of the population health status and finding possible causes leading to the severe decline.

## Methods

**Fig. 1:**
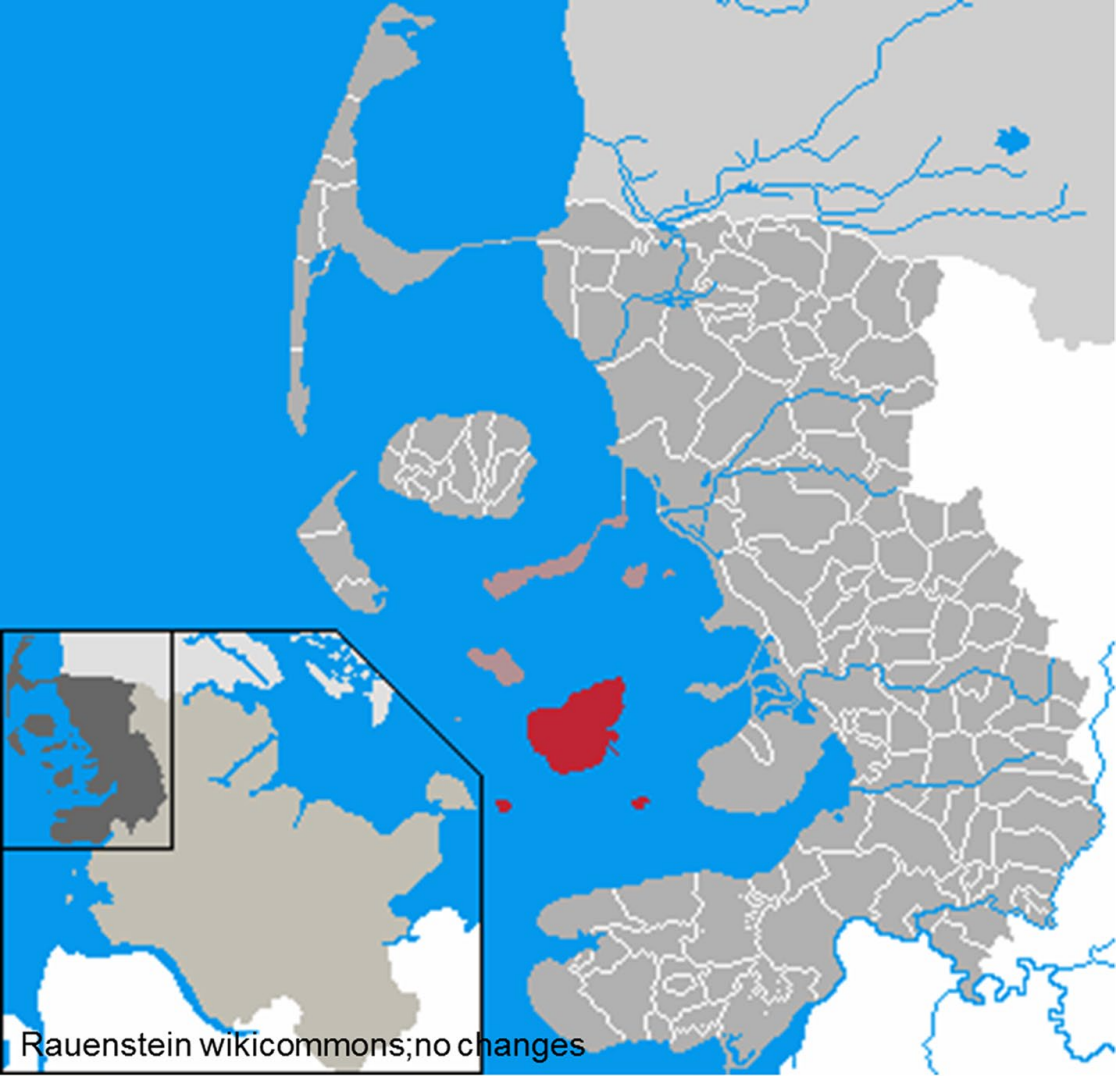
Map of northern Germany. Municipality Pellworm (*red*) in the district of Nordfriesland, federal state of Schleswig-Holstein.

Hares (n = 110) were sampled on the German North-Sea island Pellworm (54°31′N, 8°38′E; Fig. 1) in the years 2010 and 2011. The sampling was carried out during the usual two consecutive hunting seasons in December 2010 and October 2011. The hares were shot by local hunters and straight thereafter collected one-by-one, making sure no animal was shot wounded and left to die. Necropsy and sampling for histopathology, bacteriology and parasitology was performed on site 1–3 h after death. Samples for further analysis were taken when appropriate.

### Necropsy and histopathology

Specimens of all organs (heart, lung, liver, spleen, kidneys, small intestine, large intestine, mesenterial lymphnode, brain) were fixed in 7% neutral buffered formalin, processed, embedded in paraffin wax, cut at 3 µm, mounted on glass slides and stained with haematoxylin and eosin (H&E) according to standard procedures. In addition, special histological staining methods were used depending on microscopic findings, i.e. for the detection and confirmation of amyloid (Congo red staining), acid fast bacteria (Ziehl–Neelsen staining), or fungi (Grocott’s Gomori methenamine silver nitrate staining). Furthermore, when needed, immunohistochemistry (IHC) (i.e. for *Encephalitozoon cuniculi*) was performed using an established protocol [15]. Moreover the placental-scars in the uteri of the females were stained making use of the Tirmann Schmelzer reaction with Turnbull’s blue and counted according to Bray et al. [16].

### Bacteriology and parasitology

As sampling progressed, it was noted that all of the hares showed catarrhal enteritis affecting the entire small intestine, so it was decided to routinely sample them for bacteriology. In 85 hares, 40 in 2010 and 45 in 2011, samples of the small intestine (duodenum) were therefore taken for bacteriologic screening. The isolation of bacteria from small intestine samples was performed as follows: with a sterile swab intestinal contents were platted into MacConkey II agar (MC), Columbia CNA Agar with 5% sheep blood, improved II (CNA), two BD Columbia III Agar with 5% sheep blood (BA), BD Campylobacter Bloodfree Selective Medium (Campy) and BBL™ Sabouraud Dextrose Agar with chloramphenicol and gentamicin (SAB) (all from Becton–Dickinson, Heidelberg, DE). MC, CNA and one BA were incubated at 37°C for 24 h. Furthermore one BA was incubated at the same conditions in an anaerobic atmosphere. Campy was incubated at 42°C for 48 h in microaerobic atmosphere. For selective isolation of β-lactamase producing *Enterobacteriaceae* intestinal content was precultured at 37°C overnight in buffered peptone water (BPW) (Merck, DE) supplemented with cefotaxime (1 mg/l) and then cultivated at 35°C overnight on McConkey agar (MCA) (Oxoid, Basingstoke, UK) supplemented with cefotaxime (1 mg/l), which select for broad-spectrum-cephalosporin-resistant isolates. For the isolation of plasmid-mediated quinolone resistant (PMQR) intestinal content was precultured at 37°C overnight in BD MacConkey Broth (Becton–Dickinson, Heidelberg, DE) and then cultivated at 35°C overnight on MCA supplemented with 0.06 mg/l ciprofloxacin. For selective isolation of *Salmonella* sp. intestinal samples were inoculated into 9 ml Rappaport–Vassiliadis enrichment broth (Oxoid, Vienna, AT) and 9 ml selenite cysteine bouillon (Oxoid, Vienna, AT) and incubated at 42°C. After 24 h one loopful of enrichments cultures were streaked onto BD XLD Agar (Becton–Dickinson, Heidelberg, DE) and incubated at 37°C for 24 h. Identification of bacteria was performed on the basis of phenotypic characteristics.

In two cases further bacteriological analysis of frozen (−80°C) tissue (mesenterial lymphnodes) was performed as described above, after lesions were recognized by histopathology, as well as a PCR for the detection of *Mycobacteria* [17].

All hares were screened for *Francisella tularensis* by culture of frozen (−80°C) tissue samples as reported elsewhere [18]. If pathologic changes suspicious of bacteriologic infection were noted in other organs, additional bacteriologic analyses were performed of these samples.

For parasitology, a flotation of faecal samples was performed. The nematode burden of the entire gastro-intestinal tract was counted according to procedures explained elsewhere [19]. Furthermore a quantitative assessment of coccidia was performed using the McMaster method [20].

### Serology

Post morten blood samples for serology were collected using 4 ml Z Serum Sep Clot Activator tubes (Greiner BioOne, AT). The serum was centrifuged, separated and frozen at −20°C until further processing. EBHS serology was performed on 32 hares in 2011 using an established competitive enzyme linked immunosorbent assay (ELISA), highly specific for EBHSV [21, 22]. The serologic screening for *Treponema* sp. was performed on 41 hares in 2011. For this an indirect immunofluorescent test (IFAT) was set up [23].

### Statistics

Being binomial datasets the sero-prevalences and confidence intervals (CI 95%) for EBHS and *Treponema* sp. were calculated as described by Hald [24] using microsoft excel.

## Results

Altogether 110 European brown hares were sampled in the years 2010 and 2011. Fifty-eight hares were sampled in December 2010; 52 in October 2011. Animals were divided into two age classes: adult and sub-adult, using lens-weight [25] and the sign of “Stroh” [26]. Palpating the sign of “Stroh” means looking for the epiphyseal cartilage plate of the lower extremity and feeling the ossification of the epiphysis plate of the ulna/the epiphyseal protrusion of the ulna. This yielded 44 adult females, 18 sub-adult females, and 29 adult and 19 sub-adult males. The percentage of sub-adult animals was 34 and 32% for 2010 and 2011 respectively. The body weight ranged from 1,209 to 4,685 g. Twenty uteri were examined for placental scars in 2010, and a maximum of 15 placental scars were found, with a mean of 8.6. In 2011 28 uteri were examined and a maximum of 13 scars were found, with a mean of 7.5. Solely one uterus showed pathological changes (several cysts) and was sampled for further bacteriological analyses. Only in three animals in 2010, and two in 2011 pathologic changes were absent. All other animals (n = 55 in 2010, n = 50 in 2011) showed lesions in at least one organ.

### Necropsy findings

**Fig. 2:**
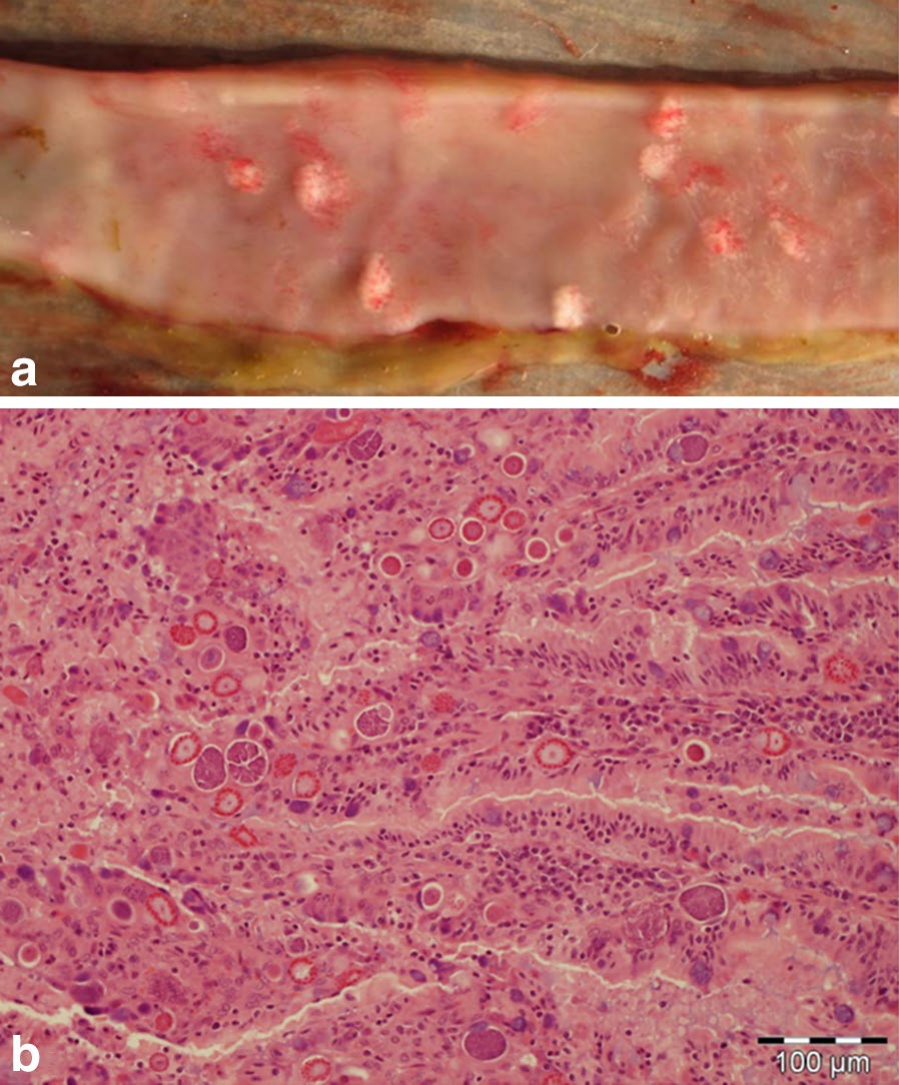
**a** Part of the small intestine with multifocal raised white nodules representing aggregates of coccidia. **b** Photomicrograph of small intestine with various stages of coccidia in the lamina propria. H&E; *bar* 100 µm.

**Fig. 3:**
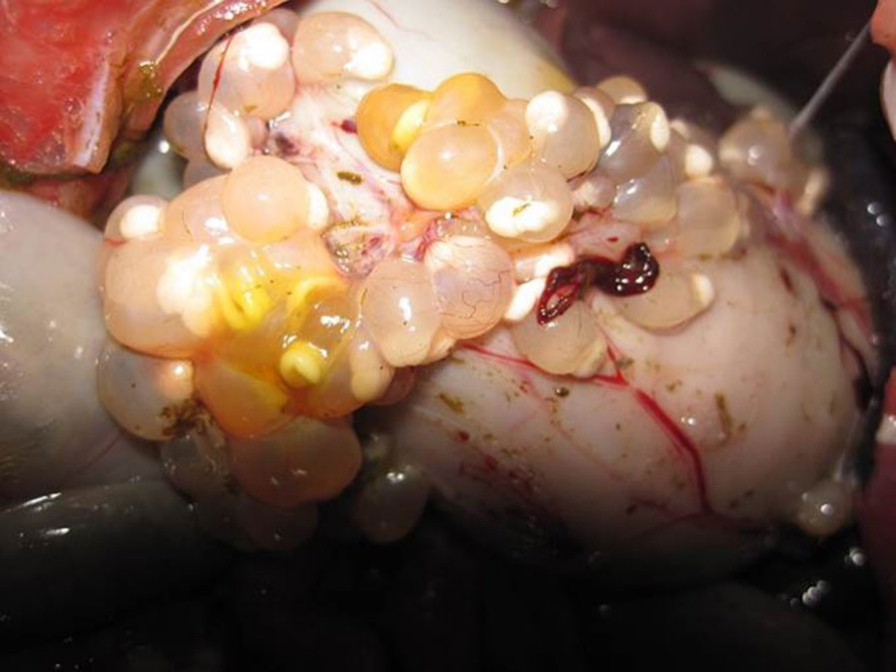
Multifocal cysts of *Taenia pisiformis*, *Cysticercus pisiformis*, attached to the serosa of the stomach and intestine.

**Fig. 4:**
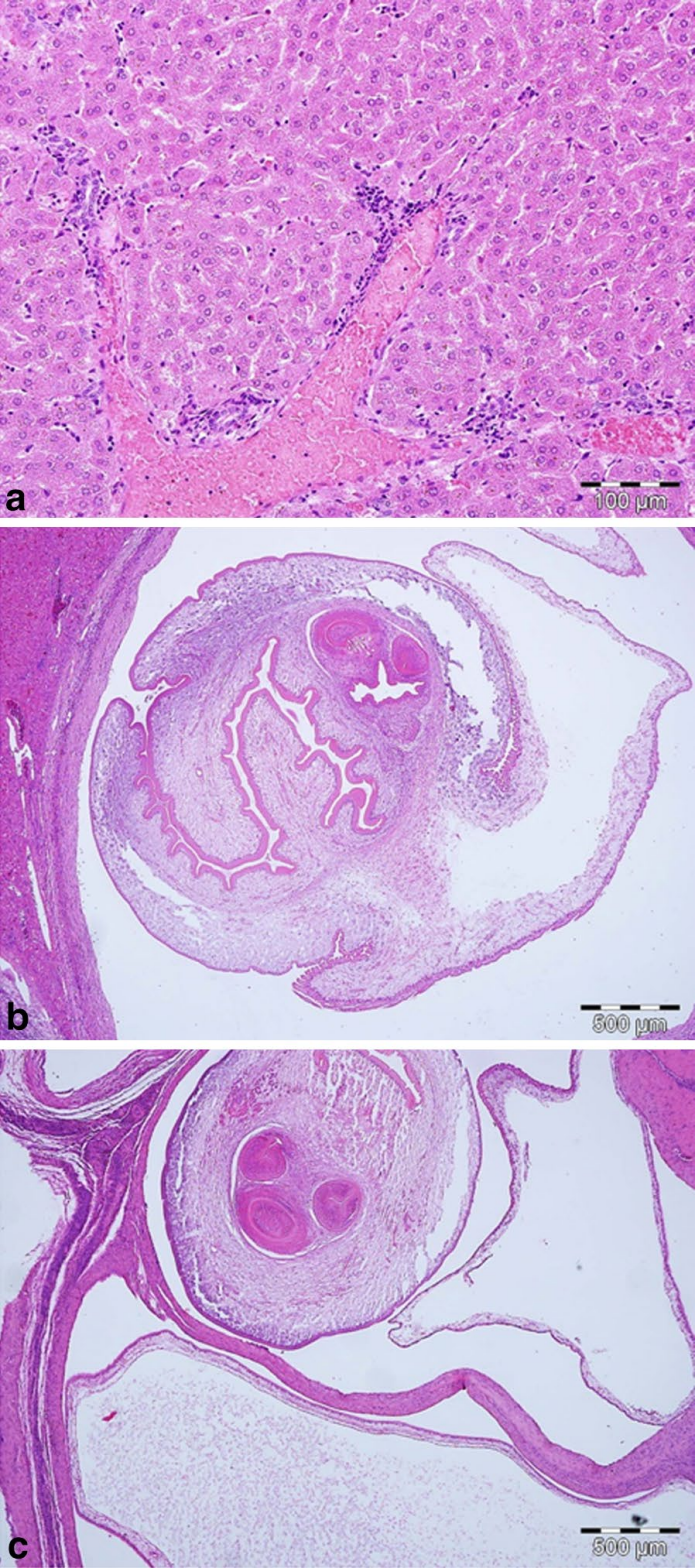
**a** Mild lymphoplasmacytic inflammatory infiltration surrounding a portal tract in the liver. H&E; *bar* 100 µm. **b** Parasitic cyst of *Cysticercus pisiformis* surrounded by a dense fibrous capsule infiltrated mostly by lymphocytes and plasma cells compressing the liver parenchyma. H&E; *bar* 500 µm. **c** Parasitic cyst of *Cysticercus pisiformis* separated by bands of fibrosis and a mostly lymphoplasmacytic inflammatory reaction in the mesentery. H&E; *bar* 500 µm.

The overall nutritional state of the animals was good. The main lesions found during both sampling periods was a catarrhal enteritis affecting the entire small intestine, and in 27 cases (24.5%) gross lesions produced by intestinal coccidia, i.e. multifocal raised white nodules (Fig. 2a), were seen. Furthermore only a small number of animals had solid faeces. In eight animals cysts of a tapeworm were found. These were later confirmed to be cysts of the tapeworm *Taenia pisiformis,* namely *Cysticercus pisiformis*. The cysts were mainly found in proximity to the intestinal tract (Figs. 3, 4), but in two cases they could be found in the thoracic cavity.

Additionally single disease incidents were found during necropsy. These included an approximately walnut-sized abscess in the mammary gland area of one adult female hare, a suppurative bronchitis in a sub-adult male hare, and a moderate pyometra with multiple cysts in an adult female hare. These altered organs were sampled for bacteriology.

### Histopathological results

#### Gastrointestinal tract

The main determined lesion was a moderate to severe chronic lympho-plasmacytic enteritis, observed in 63 hares (32 male, 31 female; 63.0%). In 49 (23 male, 26 female; 49.0%) cases intralesional coccidia could be demonstrated (coccidia stages—micro- and macrogametes; Fig. 2b). In 22 cases (11 males, 11 females; 22.0%) intraluminal intestinal nematodes were seen. In 10 cases (three males, seven females) intestinal samples were not available for analysis.

#### Parenchymal organs

In 26 hares (13 males, 13 females; 23.6%) a moderate, multifocal, periportal, lympho-plasmacytic hepatitis was observed (Fig. 4a). Other changes in the liver included a moderate multifocal suppurative hepatitis (two males, nine females; 10.0%), massive multifocal to coalescing granulomatous hepatitis with intralesional parasites (parts of *Cysticercus pisiformis*) (three males, one female; 3.6%; Fig. 4b), and in two cases (one male, one female; 1.8%) biliary duct adenomas.

In 14 hares (five males, nine females; 12.7%) a multifocal mild to moderate chronic interstitial nephritis could be seen.

No pathological changes were noted in the heart except a mild multifocal lymphocytic myocarditis in one male animal (0.9%). Moderate multifocal suppurative pneumonia was noted in three hares (two males, one female; 2.7%).

#### Lymphatic organs

**Fig. 5:**
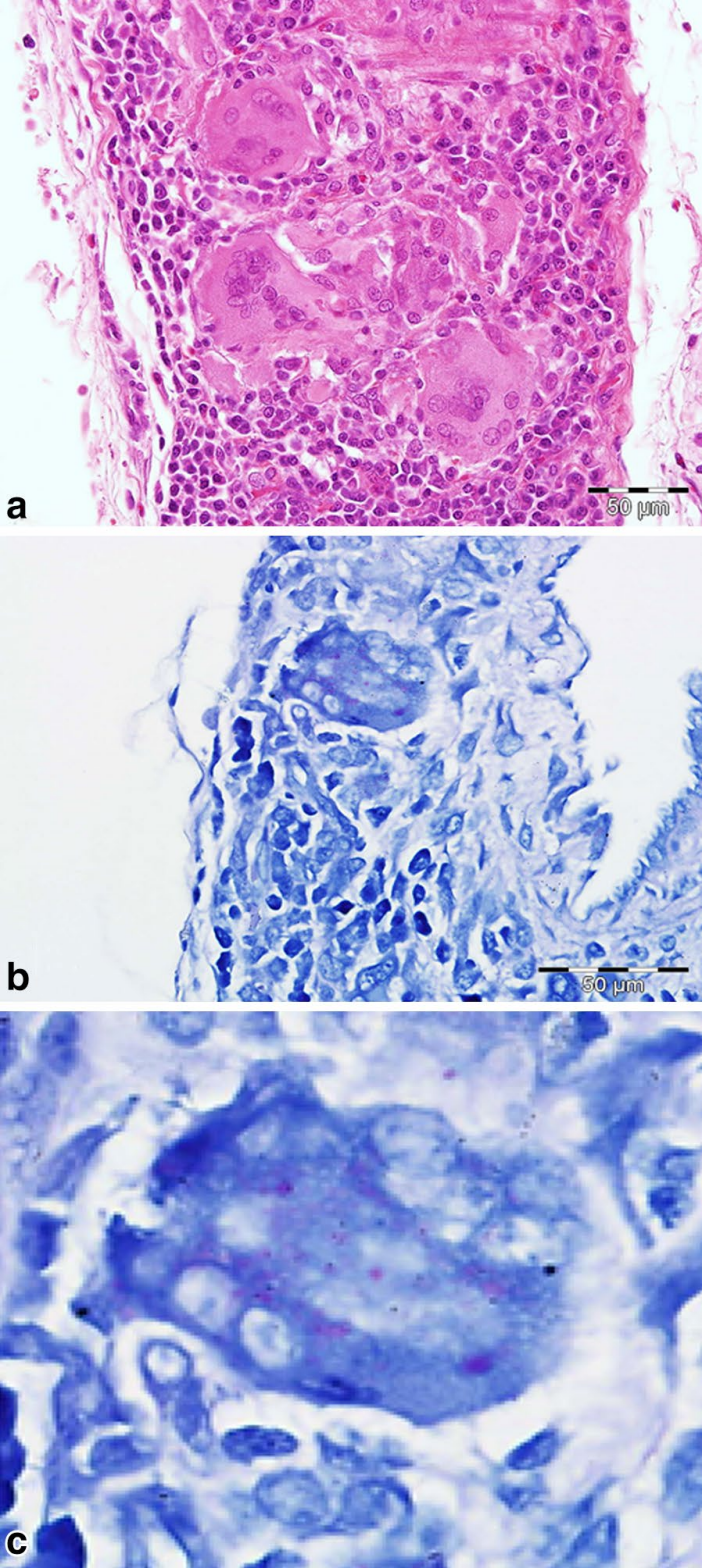
**a** Several foamy looking histiocytes as well as multinucleated giant cells are present in and around a vein of a lymph node. H&E; *bar* = 50 µm. **b** Same vein as in A with positive (*red*) material in the cytoplasm of a multinucleated giant cell. Modified Ziehl–Neelsen; *bar* 50 µm. **c** Higher magnification of the multinucleated giant cell. Modified Ziehl–Neelsen.

A moderate diffuse suppurative splenitis was found in four hares (one male, three females; 3.6%). Germinal centres were classified as active in 11 animals (three males, eight females; 10%). Five hares (two males, three females; 4.5%) had a moderate diffuse suppurative lymphadenitis. Four hares (three males, one female; 3.6%) (all 2011) had a massive multifocal to coalescing histiocytic infiltration, as well as multinucleated giant cells in the mesenterial lymphnodes. This infiltration also surrounded vessels in close proximity to the nodes (Fig. 5a). As the histiocytes and multinucleated giant cells showed a foamy, intracellular material in H&E staining, several stains, such as Gram, Giemsa, Ziehl–Neelsen and modified Ziehl–Neelsen were performed. The modified Ziehl–Neelsen staining showed acid-fast elements (Fig. 5b, c). However to date all attempts to identify *Mycobacteria* using PCR proved negative. A further bacteriological analysis of frozen (−80°C) tissue was performed and yielded *Escherichia coli*, *Staphylococcus* sp. and Gram-positive cocci.

#### Brain

The microsopridia *Encephalitozoon* was demonstrated by IHC in the brain of three hares (two males, one female; 2.7%) showing a moderate multifocal lympho-plasmacytic to granulomatous encephalitis.

### Bacteriology and parasitology

Eighty-five samples of small intestine (40 in 2010, 45 in 2011) underwent bacteriologic examination. In all samples a small proportion of Gram-positive aerobic endospore-forming rods, *Enterococcus* spp., Gram negative non-*Enterobacteriaceae*, filamentous fungi and in rare cases *Saccharomycetales* were detected. None of the additionally targeted bacteria (i.e. *Clostridium* sp., *Campylobacter* sp., *Salmonella* sp., β-lactamase producing *Enterobacteriaceae* and PMQR) could be isolated. In 2010 a high-grade predominance of *E. coli* was found in the intestine of nine animals and in 24 hares from 2011. In six animals in 2010 the intestinal flora had a high grade predominance of *Aeromonas* sp, whereas none of the animals had a high predominance of this bacterium in 2011. The numbers of double infection (high dominance of these two bacteria) in 2010 and 2011 was 22 and 16 respectively. The detailed bacteriologic results of the intestinal samples are summarized in Table 1. Due to noted macroscopic changes, the following additional samples were collected for bacteriology—one lung, which yielded a Gram-negative mixed flora; one uterus, yielding Gram negative non-*Enterobacteriaceae* and one abscess (mammary gland area) demonstrating *E. coli* and *Staphylococcus aureus*. The *S. aureus* strain was analysed in depth and proved to be the first known isolate of a hare carrying a *mecC*-positive MRSA [27]. All hares tested negative for *F. tularensis.* This result however has to be evaluated with care, as the samples were evaluated using a new method in the course of another ongoing project. However necropsy findings of tularemia was not observed in any of the hares.

**Table 1:** Results of the bacteriologic analysis of samples of small intestine (n = 85) from hares for the years 2010 and 2011

	2010	2011
High predominance *E. coli*	9 (22.5%)	24 (53.3%)
High predominance *Aeromonas* sp.	6 (15%)	0
Predominance aerobic spore-forming bacteria	1 (2.5%)	1 (2.2%)
*E. coli* and *Aeromonas* sp.	22 (55%)	16 (35.6%)
*E. coli* and *Aeromonas* sp. and *Staphylococcus* sp.	0	1 (2.2%)
*E. coli* and *Staphylococcus* sp.	0	2 (4.4%)
*E. coli* and anaerobes and *Klebsiella* sp.	0	1 (2.2%)
*Aeromonas* sp. and mucoid *E. coli*	1 (2.5%)	0
*E. coli* and anaerobes	1 (2.5%)	0

The parasitic infestation varied highly. No lung worms or gastric parasites were found. The most abundant parasite was *Eimeria* spp. Coccidia were found, by the flotation method, in all but seven of the tested animals. Using the McMaster counting chamber a mean of 4,867 oocysts/g faeces (maximum 100,000–minimum 200 oocysts/g faeces) in 2010, and a mean of 2,902 oocysts/g faeces (maximum 17,100–minimum 100 oocysts/g faeces) in 2011 were counted. In six hares (four in 2010; two in 2011) no nematodes (i.e. *Trichostrongylus retortaeformis*, *Trichuris* sp.) were detected. The maximum number of *T. retortaeformis* counted in one hare was 12,020. This hare was a female adult hare of good nutritional state, but with a very high load of *E. coli* and *Aeromonas* sp. in the intestine.

### Serology

The sero-prevalence for EBHS investigated in 32 hares was 78.1% (CI 62.95–92.14%; n = 25). Fourteen adults, 4 subadult females and 7 adult males were positive. The overall sero-prevalence for *Treponema* sp. investigated in 41 hares was 43.9% (CI 28.82–60.1%; n = 18). Significantly more adult females (n = 15) tested positive for *Treponema* sp. Solely one sub-adult female, and two adult males were positive [23].

## Discussion

Possible causes of single deaths and a declining population in hares and generally other species are many; reaching from obvious causes like diseases [28] and predation [29], to climate changes [30], habitat loss [31] and the cost of reproduction [32, 33]. Therefore identifying the cause of a decline event necessitates multidisciplinary approaches. To get an overview of the overall health status of the population a thorough health assessment program was implemented to elucidate pathologic causes for the sudden and still ongoing decline of the local European brown hare population.

Bray et al. [16] showed that each doe can produce up to 15 (5–15) leverets per season. Although it has been discussed that the reproductive success may suffer from agricultural practices [34], the results of our study with a mean of 8.5 (maximum 15), and 7.4 (maximum 13) placental scars in 2010 and 2011 respectively, indicate a solid reproduction. Moreover, the local hunters note numerous young hares in spring and summer, but in autumn and winter these animals are suddenly missing. This was already shown in 2007/08, when 30–50% of the hare population was missing at the annual count in autumn (pers. comm. Mr. Ewers, Dr. Hoffmann). The red fox (*Vulpes vulpes*) is known to cause high mortality rates in hares, especially if the density of foxes is high in an area [9, 35]. Being an island free of predators, except for approximately 12 resident Western marsh harrier (*Circus aeruginosus*) breeding pairs and European herring gulls (*Larus argentatus*) [36], predation as a cause for the decline was excluded from the beginning.

What is more difficult to analyze and understand is the intestinal flora of the lagomorphs, how it changes when the animal is diseased and how the changing environment influences the flora, as knowledge is scarce. However, the most striking result of this study is the variable high endoparasitosis, the catarrhal enteritis and the presumed shift in the intestinal flora. Ducluzeau et al. [37] analyzed the microflora of captive young hares in their facility in France, and found that neither *E. coli*, nor *Staphylococcus* sp. or *Lactobacillus* sp. were present in healthy young hares. Unfortunately this study only included captive leverets up to the age of weaning. Whitney [38] showed *E. coli* to be present in the large intestine of rabbits, but not in the small intestine. Our samples consisted of small intestine, due to the noted catarrhal enteritis. In our own experience, at least in captive hares, *E. coli* is not a part of the normal flora regardless the age (unpublished data). In our own dataset of free-ranging hares from Austria *E. coli* seems to play a minor role in the microflora (unpublished data). The main difference between the Austrian hares and the hares of this study is the high predominance of one or two bacterial species in the culture, namely *E. coli* and *Aeromonas* sp. In healthy animals in general, the microflora is markedly more diverse in the intestinal tract. The results of this study point towards a shift and a decreased diversity in the microflora, which can lead to ill-thrift and a reduced function of the immune system [e.g. 39, 40].

The combination of the postulated shift of the intestinal flora and the high parasitic burden inevitably leads to a reduction in fitness, due to a compromised function of the intestine. The hares in our study showed a high infestation with *Eimeria* spp., as well as intestinal nematodes. Macroscopic lesions produced by *Eimeria* spp. were found in 24.5% of the hares. The underlying histopathological changes of the intestine may induce malabsorption, anaemia, hypoproteinemia and dehydration [41]. Although parasite infestation often occurs without clinical effects in wildlife, a severe infestation can lead to a reduced fitness of the host [42]. Coccidia are one of the most potent pathogenic parasites in hares, and the combination of intestinal nematodes and coccidia has been shown to be one of the major regulatory factors in hare populations [43]. Especially in young animals a high level of parasitic infestation can lead to ill-thrift and subsequently to the death of the animal.

Except for a moderate hepatitis and histiocytic lymphadenitis, lesions found in other organs do not seem a likely cause for this decline, and are in the authors’ opinion single individual events. The aetiology of the hepatitis remains unclear, but could be secondary to the enteritis since the liver is the first organ of defence to pathogens penetrating the gut mucosa [44], or be a result of other random inflammatory processes. The above mentioned changes in the lymph node (Fig. 5) are that of a chronic lymphadenitis caused by an acid-fast bacterium, which so far has not been able to be classified in detail. Furthermore, no known pathogen capable of causing an epidemic could be found. Despite the high sero-prevalence of EBHS (78.1%) and *Treponema* sp. (48.3%), there were post mortem signs of the diseases which confirm the chronic-endemic nature of the diseases, as reported across Europe [45, 46]. This is also in agreement with the model explaining the natural diffusion of EBHS in relation to variable hare population densities, which suggests the existence of high EBHSV prevalence when densities are over 15 individuals/km^2^ [22, 47].

It has been well established that the agricultural situation of the island has gone through severe changes in the last decade towards a monoculture of corn-production for biogas plants (pers. comm. Mr. Ewers). In his assessment of the habitat of hares in Switzerland Baumann [48] showed that hares have clear preferences and actively avoid certain types of fields/structures. Fields with a vegetation height of under 15 cm are avoided, as are harvested and harrowed fields. Flowered fallows and fields with vegetation of approximately 20–35 cm height are preferred. Moreover, Smith et al. [4] postulate that agricultural intensification and global climate change are two of the main causes suspected to be responsible for a decline in hares. Additionally, the implementation of biogas plants on the island has led to an increase in harvesting and manuring frequency of the fields. This intensification of agriculture not only leads to a loss of habitat, but also to more hares lost to the tilling process, and potentially engenders a higher pathogen/bacterial load (i.e. *E. coli*) in the environment due to the increased manuring. This combined with other stressors as e.g. bad weather conditions (e.g. rain) could explain the increased susceptibility towards bacteria/parasites which in a normal scenario might not have such fatal consequences.

## Conclusions

The changes in the intestinal tract could have led to the observed decline in the hare population, but further in depth research is needed to elucidate the composition of the hare’s microflora and how these presumed changes were caused. If and how a change in the habitat can lead to such a severe shift in the physiology of wildlife must be investigated further to prevent population declines as described here.

## Abbreviations

EBHS: European brown hare syndrome
ELISA: enzyme linked immunosorbent assay
H&E: haematoxylin and eosin
IFAT: immunofluorescent test
IHC: immunohistochemistry
PCR: polymerase chain reaction
